# TIP60/KAT5 is required for neuronal viability in hippocampal CA1

**DOI:** 10.1038/s41598-019-50927-1

**Published:** 2019-11-07

**Authors:** Inga Urban, Cemil Kerimoglu, M. Sadman Sakib, Haifang Wang, Eva Benito, Christina Thaller, Xunlei Zhou, Jun Yan, André Fischer, Gregor Eichele

**Affiliations:** 1 Genes and Behavior Department, Max Planck Institute for Biophysical Chemistry, 37077 Göttingen, Germany; 2 Department of Epigenetics and Systems Medicine in Neurodegenerative Diseases, German Center for Neurodegenerative Diseases (DZNE) Göttingen, 37075 Göttingen, Germany; 3 Institute of Neuroscience, State Key Laboratory of Neuroscience, CAS Center for Excellence in Brain Science and Intelligence Technology, Shanghai Institutes for Biological Sciences, Chinese Academy of Sciences, Shanghai, 200031 China; 4 University of Chinese Academy of Sciences, Beijing, China; 5 Department for Psychiatry and Psychotherapy, University Medical Center Göttingen, 37075 Göttingen, Germany; 6 Present Address: European Molecular Biology Organization (EMBO), 69117 Heidelberg, Germany; 7 Present Address: Institute of Anatomy and Cell Biology, University of Heidelberg, 69120 Heidelberg, Germany

**Keywords:** Epigenetics, Cell death in the nervous system, Epigenetics in the nervous system

## Abstract

Aberrant histone acetylation contributes to age-dependent cognitive decline and neurodegenerative diseases. We analyze the function of lysine acetyltransferase TIP60/KAT5 in neurons of the hippocampus using an inducible mouse model. TIP60-deficiency in the adult forebrain leads within days to extensive transcriptional dysfunction characterized by the presence of a neurodegeneration-related signature in CA1. Cell cycle- and immunity-related genes are upregulated while learning- and neuronal plasticity-related genes are downregulated. The dysregulated genes seen under TIP60-deficiency overlap with those in the well-characterized CK-p25 neurodegeneration model. We found that H4K12 is hypoacetylated at the transcriptional start sites of those genes whose expression is dampened in TIP60-deficient mice. Transcriptional dysregulation is followed over a period of weeks by activation of Caspase 3 and fragmentation of β-actin in CA1 neurites, eventually leading to severe neuronal loss. TIP60-deficient mice also develop mild memory impairment. These phenotypes point to a central role of TIP60 in transcriptional networks that are critical for neuronal viability.

## Introduction

Lysine acetylation is a posttranslational modification, which regulates diverse protein characteristics, including stability, subcellular localization, catalytic activity and protein interactions. Acetylation is carried out by lysine acetyltransferases (KATs) and counteracted by lysine deacetylases (KDACs). Acetylation of histones positively correlates with transcription^1–4^ and is involved in the encoding of new memories in neurons^5–8^. Deregulation of histone acetylation, for example hypoacetylated histone H4 at lysine 12 (H4K12ac), has been implicated in impaired synaptic plasticity, dementia and neurodegenerative disorders^4,9–12^. KATs are grouped into GNAT, p300/CBP and MYST families^13^. We recently compared the expression of all KATs in the hippocampal CA1 region in adult mice^14^ and found *Kat2a*/*Gcn5* and *Kat5*/*Tip60* as the two most strongly expressed KATs in this brain region. Loss of GCN5 from neurons of the adult forebrain led to specific memory impairment in mice and was also linked to the regulation of neuroactive ligand-receptor signaling associated gene expression programs^14^. The second most strongly expressed KAT, *Tip60*, was discovered as a TAT-interactive protein 60 kDa (TIP60) and is a MYST family member, characterized by a 300 amino acid MYST domain that comprises an acetyl-CoA binding catalytic domain and a zinc finger^15^. TIP60 is the catalytic subunit of the evolutionarily conserved NuA4 complex^16^. This complex has been implicated in multiple cellular processes, including DNA damage repair, apoptosis, mitosis and cancer^17–20^. Histone H4 is a major TIP60 substrate^17^. In addition to histones, TIP60 acetylates transcription factors and also functions as coactivator or corepressor (reviewed in ref.^21^). This multifaceted mode of action designates TIP60 as a hub of gene expression regulation.

Despite a wealth of biochemical data, little is known about the physiological function of mammalian TIP60 in the adult brain because of early embryonic lethality of TIP60-deficient mice^22^. To gain insight into the function of TIP60 in the mammalian brain, we have generated a conditional *Tip60* knockout mouse line and induced TIP60-deficiency in postmitotic excitatory neurons of the adult forebrain using a tamoxifen-inducible driver line^23^. Within 10 days after *Tip60* deletion, we found a massive dysregulation of gene expression in the hippocampal CA1 region, concurrent with a significant reduction of H4K12 acetylation at transcription start sites of downregulated genes in *Tip60* conditional KO mice. Already 3 weeks after *Tip60* deletion we observed scarce neurodegenerative processes that eventually led to progressive neuronal loss in CA1.

## Results

### Efficient deletion of *Tip60* in the mouse hippocampus of adult mice

**Figure 1:**
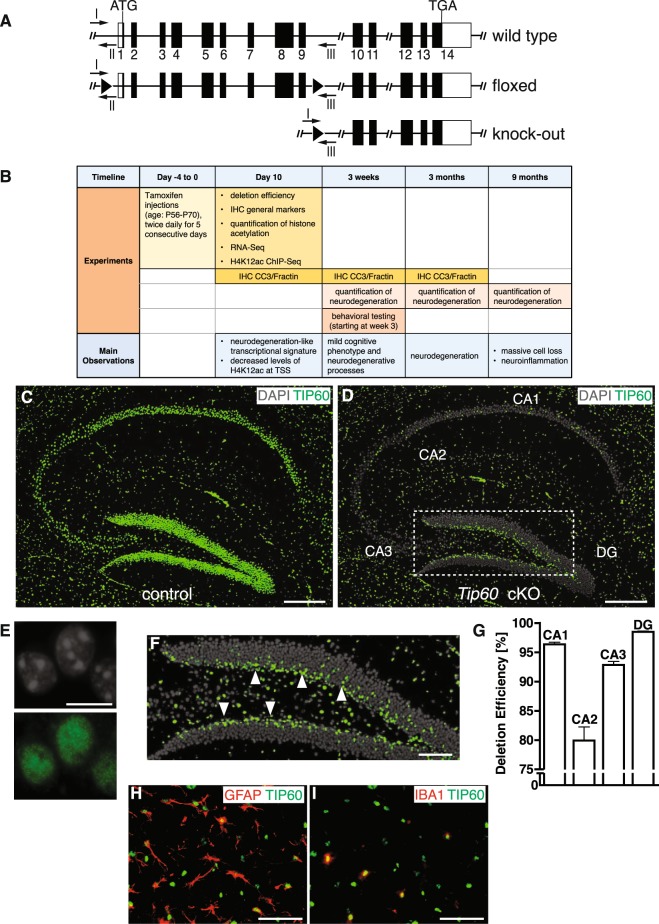
Deletion of *Tip60* in excitatory forebrain neurons of adult mice. (**A**) *Tip60* alleles for wild type, floxed, and knock-out. Protein coding exons are shown in black. LoxP sites are indicated with black triangles. Genotyping primers I, II and III are indicated with arrows. (**B**) Time points when experiments were conducted and main observations. The last day of tamoxifen injections is defined as day 0. (**C**) Ubiquitous nuclear TIP60 signal in the hippocampus of control mice at day 10 after the last tamoxifen injection. (**D**) In *Tip60* cKO most of the TIP60 signal in the principal cell layers is absent at day 10 after the last tamoxifen injection. (**E**) TIP60 signal is nuclear. TIP60 and DAPI staining in single hippocampal nuclei are shown. (**F**) Marked area from (**D**) showing TIP60-positive nuclei in the subgranular zone (arrowheads), a region lacking CRE activity. (**G**) Quantification of deletion efficiencies in CA1, CA2, CA3 and DG in *Tip60* cKO mice at day 10 after tamoxifen injections, normalized to the total number of DAPI positive nuclei (n = 4, 4 sections per animal). Error bars represent SEM. (**H**,**I**) Pictures of triple labeling of TIP60, GFAP and IBA1 in the *stratum lacunosum moleculare* of the CA1 region in a *Tip60* cKO mouse. (**H**) Shows TIP60 and GFAP, (**I**) TIP60 and IBA1. Scale bars: 250 µm (C,D), 10 µm (E), 100 µm (F), 50 µm (H,I). Abbreviations: CA1, hippocampal subfield CA1; CA2, hippocampal subfield CA2; CA3, hippocampal subfield CA3; DG, dentate gyrus; IHC, immunohistochemistry; CC3, cleaved Caspase 3.

To study the function of TIP60 in the adult mouse hippocampus, we crossed mice carrying a floxed *Tip60* gene (Fig. 1A) with the tamoxifen-inducible *CaMKCreER*^*T2*^ driver line^24^. This driver directs gene deletion to postmitotic excitatory neurons of the forebrain including those in the hippocampus. At 8 to 10 weeks of age both *CaMKCreER*^*T2*^ *Tip60*^*f/f*^ (*Tip60* cKO) and *Tip60*^*f/f*^ (control) mice were repeatedly injected with tamoxifen (Fig. 1B). We define the last day of tamoxifen injections as day 0.

At day 10, we performed immunohistochemistry with a custom-made TIP60-specific antibody (TIP60P4) and detected nuclear expression of TIP60 protein in the principal cell layers of all hippocampal subregions in controls (Fig. 1C,E). Hippocampi of *Tip60* cKO mice showed strong reduction in the number of TIP60 expressing cells in CA and dentate gyrus regions by day 10 after the last tamoxifen injection (Fig. 1D,F). TIP60 deletion efficiency was >90% in the principal layers of the hippocampus except for the CA2 region (Fig. 1G) demonstrating effective gene deletion. Because the CaMKCreER^T2^ driver is not expressed in neuronal progenitors and glial cells, TIP60 is still detected in subgranular neurons (Fig. 1F, arrowheads) and in GFAP- or IBA1-expressing glial cells (Fig. 1H,I).

The expression patterns of neuronal nuclear marker NeuN, presynaptic marker Synaptophysin 1 (SYP), dendritic marker microtubule-associated protein 2 (MAP2), and glial marker GFAP were not changed when analyzed at day 10 after tamoxifen injections (Supplementary Figure S1) suggesting that *Tip60* deletion had no immediate effect on hippocampal gross morphology.

### TIP60-deficiency leads to extensive neurodegeneration in CA1

**Figure 2:**
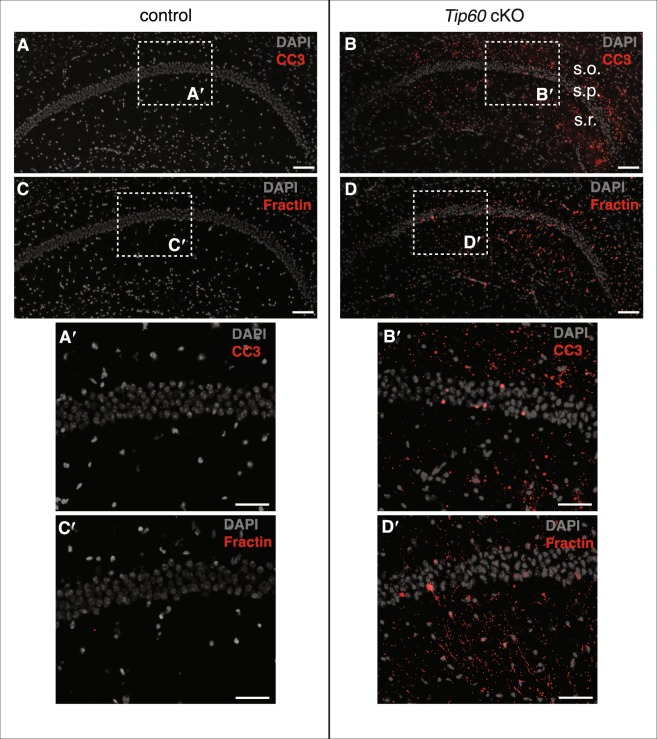
Neurodegenerative phenotypes in TIP60-deficient mice 3 months after *Tip60* deletion. (**A**) CA1 in controls lacks cleaved Caspase 3 (CC3) signal. (**B**) CC3 signal is strong in CA1 of *Tip60* cKO animals. (**C**) Absence of Fractin signal in CA1 of controls. (**D**) Fractin signal in CA1 of *Tip60* cKO mice. Sections shown for CC3 and Fractin are adjacent. (**A′**–**D′**) Enlargement of areas indicated in (A–D). In *Tip60* cKO mice CC3 and Fractin signals are mainly located in the *stratum oriens* and *stratum radiatum* with single positive nuclei in the *stratum pyramidale*. Scale bars: 100 µm (A–D), 50 µm (A′–D′). Abbreviations: CC3, cleaved Caspase 3, s.o., *stratum oriens*; s.p., *stratum pyramidale*; s.r., *stratum radiatum*.

When monitoring the brains of aging *Tip60* cKO animals histologically, we noticed a substantial age-dependent neuronal loss in the hippocampus, suggesting a neurodegenerative phenotype. We analyzed the activation of apoptotic marker Caspase 3 (CC3) and its specific cleavage product Fractin (fragments of actin, ref.^25^) at 3 different time points (Fig. 1B). By day 10, the CA1 region of *Tip60* cKO and control mice were indistinguishable. In contrast, by 3 weeks after deletion of *Tip60*, CC3 and Fractin were present, but only at minimal levels and only in the most medial part of CA1 (Supplementary Figure S2). By contrast, at 3 months, wide-spread CC3 and Fractin signals were detectable in the CA1 region of *Tip60* cKOs (Fig. 2B,D) but were absent in controls (Fig. 2A,C). CC3 and Fractin signals were highly specific for CA1 and mainly located in the *stratum oriens* and *stratum radiatum* (Fig. 2B′,D′). CC3 was also observed in single nuclei of the CA1 pyramidal cell layer of *Tip60* cKO but not control mice (Fig. 2A′,B′). The same was observed for Fractin (Fig. 2C′,D′), where we also frequently detected single neurons completely filled with Fractin signal (Supplementary Figure S3). Reminiscent of the week 3 time point, but much more pronounced, there was a graded decline of signal in a medial to lateral direction at 5 months (Supplementary Figure S3).

**Figure 3:**
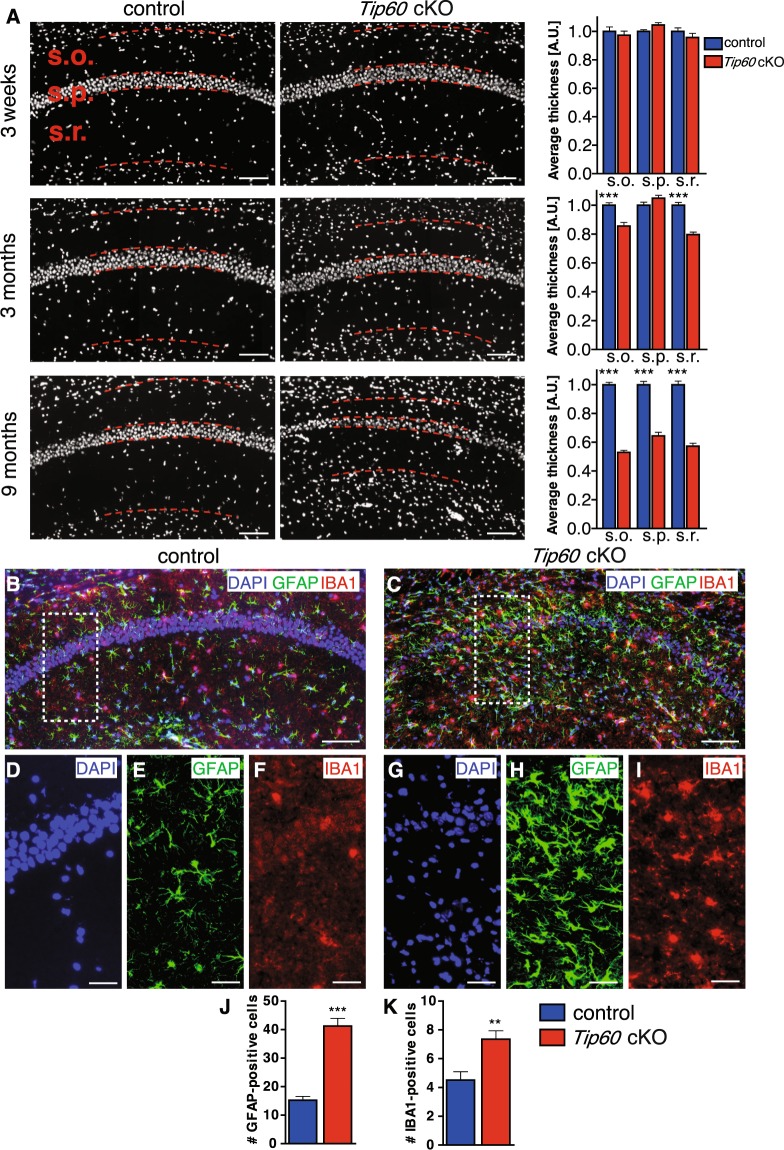
Loss of *Tip60* leads to neurodegeneration and inflammation in the CA1 region. (**A**) Average thickness of CA1 layers *stratum oriens* (s.o.), *stratum pyramidale* (s.p.), and *stratum radiatum* (s.r.) was quantified in control and *Tip60* cKO mice at 3 weeks, 3 months and 9 months after *Tip60* deletion. The borders of the layers are indicated by dashed lines. n = 2–4 per genotype, 4 sections per animal. Two-tailed student’s t-test, ***p < 0.0001. Error bars represent SEM. (**B**,**C**) Representative images of IBA1 and GFAP signal in the hippocampus of controls (B) and *Tip60* cKO mice (C) 9 months after tamoxifen injections. **(D**–**F**) The inset in (B) at higher magnification showing DAPI (D), GFAP (E) and IBA1 (F). (**G**–**I**) The inset in (C) at higher magnification showing DAPI (G), GFAP (H) and IBA1 (I). (**J**,**K**) Quantification of the number of GFAP- and IBA1-positive cells in the CA1 region of control and *Tip60* cKO animals. n = 3–5 per genotype, 4 sections per animal. 3 boxes of the size shown in (B,C) were counted per section. Two-tailed student’s t-test, **p < 0.01, ***p < 0.001. Error bars represent SEM. Scale bars: 100 µm (A–C), 50 µm (D–I).

The neurodegenerative processes revealed by CC3 and Fractin signal were accompanied by a substantial and progressive cell loss in the CA1 region that became particularly obvious at 9 months after *Tip60* deletion (Fig. 3A). We quantified the average thickness of *stratum oriens*, *stratum pyramidale* and *stratum radiatum* at 3 weeks, 3 months and 9 months after *Tip60* deletion (Fig. 1B). While there were no changes at 3 weeks, we observed a significant reduction in the thickness of *stratum oriens* and *stratum radiatum* by 14% and 20%, respectively, at 3 months. At 9 months the average thickness of *stratum oriens*, *stratum pyramidale* and *stratum radiatum* had decreased by 47%, 36% and 43%, respectively (Fig. 3A, p < 0.0001).

Since neurodegeneration is often accompanied by inflammatory processes, we performed immunohistochemistry using microglial marker IBA1 and astrocyte-specific marker GFAP in mice at 9 months after gene deletion (Fig. 3B,C). Both markers were strongly upregulated in the CA1 region of *Tip60* cKO mice (Fig. 3H,I) when compared to controls (Fig. 3E,F) suggesting significant neuroinflammation as a result of *Tip60* deletion from excitatory neurons of the adult brain. Quantification of the number of positive cells revealed an upregulation of about 3-fold for GFAP (Fig. 3J) and almost a doubling for IBA1 (Fig. 3K).

These data suggest that *Tip60* deletion progressively leads to neurodegeneration accompanied by neuroinflammation.

### Histone H4 and H4K12 acetylation levels are strongly reduced in TIP60-deficient CA1 neurons

**Figure 4:**
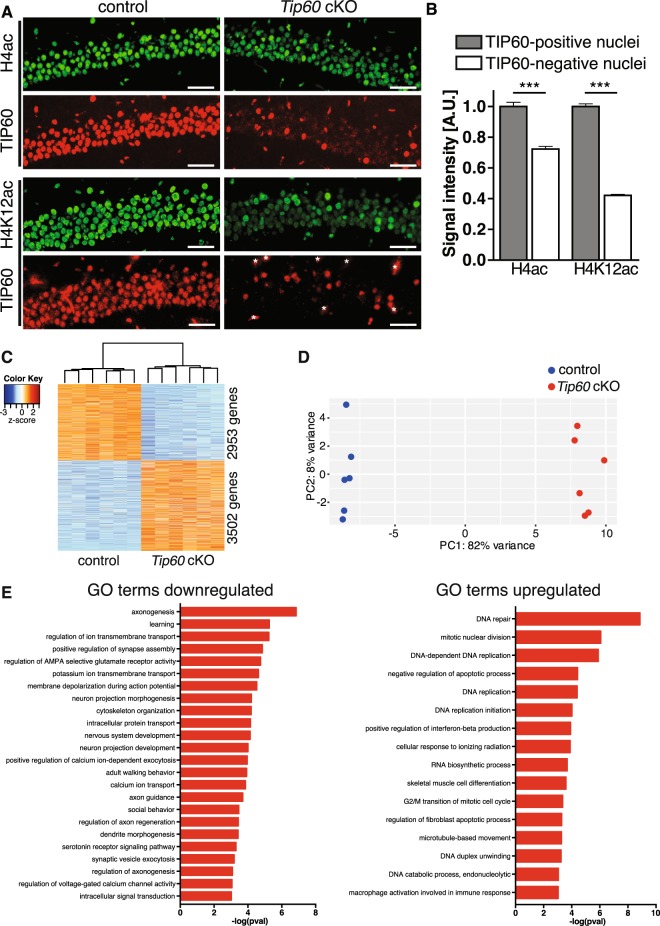
TIP60-deficiency causes reduced H4 and H4K12 acetylation and extensive dysregulation of the CA1 transcriptome. (**A**) Double labeling for TIP60 and H4ac as well as TIP60 and H4K12ac in the CA1 region of control and *Tip60* cKO mice. Sections were immunostained with antibodies against H4ac and H4K12ac (green) and against TIP60 (red) (upper panel: anti-TIP60P4; lower panel: anti-TIP60 C-7). Note the single-cell correlation between presence of TIP60 and acetylation intensity. Anti-TIP60 C-7 also stained blood vessels in both genotypes (marked by asterisks in *Tip60* cKO). (**B**) Quantification results of H4 and H4K12 acetylation signal intensities in TIP60-positive and -deficient nuclei in the CA1 region of *Tip60* cKO mice (n = 4, 4 sections per animal, >100 nuclei, two-tailed student’s t-test, ***p < 0.0001). A.U. = arbitrary units. Error bars represent SEM. Scale bars: 250 µm (A), 50 µm (B). (**C**) Heatmap depicting differential gene expression in CA1 10 days after *Tip60* deletion (n = 6 per genotype, adjusted p-value < 0.05 and |fold change| > 1.2). (**D**) Principal component analysis of gene expression profiles from the CA1 region of control and *Tip60* cKO mice shows clear separation of the two genotypes. (**E**) Genes downregulated in the TIP60-deficient CA1 region encompass learning, synaptic plasticity and neuron projection development-related functional categories, whereas upregulated ones include DNA replication, mitosis and immune response-related functional categories.

Because the neurodegenerative phenotype is highly specific for CA1 we focused our subsequent work primarily on this region. We quantified the level of acetylation of histone H4, which is a major TIP60 substrate^17^. Double labeling experiments showed ubiquitous TIP60, H4ac and H4K12ac nuclear signals across the CA1 principal cell layer in day 10 control animals (Fig. 4A, left panels). In contrast, *Tip60* cKO mice exhibited the expected reduction in the number of TIP60-positive neurons and the intensity of H4ac and H4K12ac signals was markedly reduced in many nuclei (Fig. 4A, right panels). Importantly, nuclei with reduced H4ac and H4K12ac signals were those that were TIP60-deficient. Quantification of the immunofluorescence signal showed that H4 and H4K12 acetylation were significantly downregulated in TIP60-deficient cells by 29% and 58%, respectively (Fig. 4B, p < 0.0001). The fact that H4K12ac was diminished to 42% indicated that this lysine is a major target of TIP60 also in the brain, which is consistent with previous work in other cell types^26,27^. Residual acetylation seen in *Tip60* cKO mice is probably mediated by other acetyltransferases expressed in the hippocampus (reviewed in ref.^28^). In conclusion, there is a strong molecular phenotype of hypoacetylation, especially of H4K12, in CA1 neurons, 10 days after *Tip60* deletion.

### TIP60-deficiency leads to major changes in the CA1 transcriptome

Because homeostasis of histone acetylation is essential for transcriptional regulation RNA-Seq was performed using CA1 tissue at day 10 after tamoxifen injections. Applying an adjusted p-value < 0.05 and |fold change| > 1.2 we found 3502 genes up- and 2953 genes downregulated (Fig. 4C and Supplementary Table S1). A stricter cut-off (adjusted p-value < 0.05 and |fold change| > 2) still yielded many dysregulated genes (642 up- and 168 downregulated, Supplementary Table S1). Principal component analysis (PCA) showed that the transcriptomes of *Tip60* cKO and control mice were clearly separated by the first component (Fig. 4D), underlining major differences in the transcriptomes between the two genotypes. RNA-Seq data were validated by qPCR and selected differentially expressed transcripts were analyzed by *in situ* hybridization (ISH) to reveal spatial differences in expression. Correlation between qPCR and RNA-Seq log_2_FC values was strong (Supplementary Figure S4) and ISH results were fully consistent with the findings of the other two methods. In most cases, differential gene expression identified in CA1 (e.g. *Lin7b*) extended to CA2, CA3 and the DG (Supplementary Figure S4).

Next, we assessed functional gene ontology (GO) categories enriched among genes dysregulated in *Tip60* cKOs. Downregulated genes converged to learning, synaptic plasticity and neuron projection development-related categories (Fig. 4E left). GO categories identified for upregulated genes were related to DNA repair and replication, mitosis and immune-response-related functions (Fig. 4E right). TIP60 had previously been implicated in DNA repair^17,29,30^ and mitosis^19,20^. Of note, the upregulation of immune response-related genes is most likely a secondary effect of glial cells reacting to neuronal damage.

Because TIP60 is a known co-factor of different transcription factors (TFs) we also analyzed TF enrichment in the RNA-Seq data of *Tip60* cKOs. Both up- and downregulated genes were enriched for being targets of well-known TIP60 interacting partner E2F1^31^ (q-value < 0.05 with Benjamini & Hochberg adjustment, see Supplementary Table S2). While TF enrichment in upregulated genes was almost exclusively restricted to E2F1 or related proteins the downregulated genes were significantly enriched for being targets of numerous TFs including Myc, another well- established TIP60 interacting partner^32^. Of note, *E2f1* expression is significantly upregulated by more than two-fold in *Tip60* cKO mice (adjusted p-value < 0.0001) and could thus be involved in the upregulation of genes upon *Tip60* deletion.

### Deficiency in TIP60 affects H4K12 acetylation at the TSS of downregulated genes

**Figure 5:**
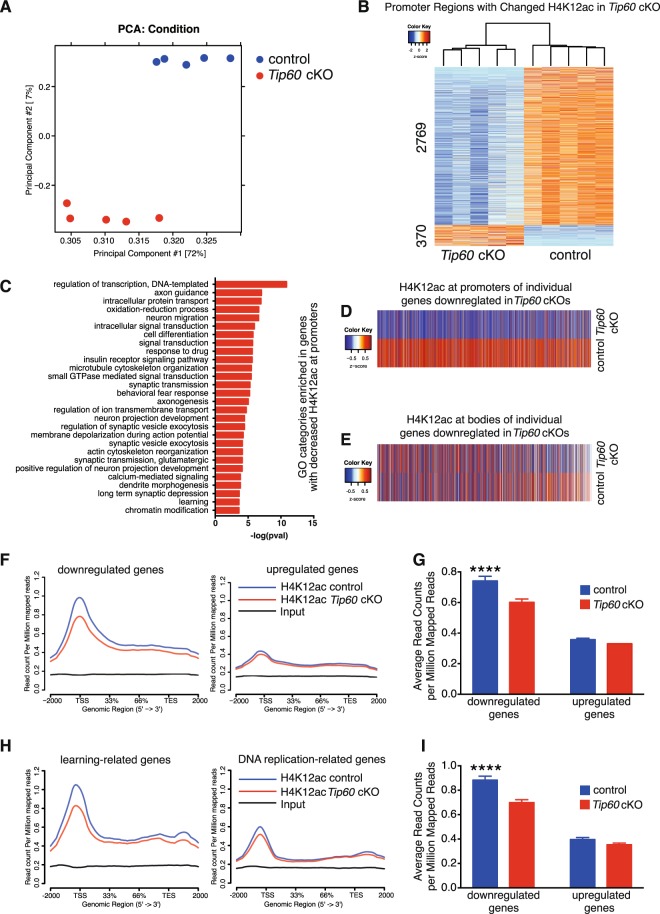
Reduction of H4K12ac binding mostly affects the promoter regions of downregulated genes in *Tip60* cKO mice. (**A**) Principal component analysis of the ChIP-Seq data shows a pronounced separation between the genotypes. (**B**) Heatmap of the promoter regions with significantly changed H4K12ac binding (adjusted p-value < 0.05 and |fold change| > 1.2). (**C**) GO category analysis for genes with decreased H4K12ac at their promoters (same genes as in (B)). (**D**,**E**) Average levels of H4K12ac at the promoters (D) and gene bodies (E) of every gene that is downregulated in the RNA-Seq analysis (adjusted p-value < 0.05 and |fold change| > 2). (**F**) H4K12 acetylation (H4K12ac) levels along the genes downregulated (left) and upregulated (right) in *Tip60* cKO mice. (**G**) Genes downregulated in *Tip60* cKO mice have significantly decreased H4K12ac levels around their TSS (+ / −2000 bp), whereas the upregulated ones do not. Moreover, downregulated genes have higher basal levels of H4K12ac compared to upregulated ones. Two-Way ANOVA: Genotype effect (control vs *Tip60* cKO): p < 0.0001; Direction effect (downregulated vs upregulated): p < 0.0001; Interaction: p = 0.0041. Post-hoc multiple comparisons for H4K12ac levels (Sidak’s multiple comparison test): downregulated genes: adjusted p-value < 0.0001 (****); upregulated genes: adjusted p-value = 0.5431. (**H**) H4K12ac levels along learning-related genes downregulated (left) and DNA replication-related genes (right) upregulated in *Tip60* cKO mice. (**I**) Learning-related genes downregulated in *Tip60* cKO mice have significantly decreased H4K12ac levels around their TSS (+ / −2000 bp), whereas DNA replication-related genes upregulated in *Tip60* cKO mice do not. Moreover, those learning-related genes have higher basal levels of H4K12ac compared to the ones related to DNA replication. Two-Way ANOVA: Genotype effect (control vs *Tip60* cKO): p < 0.0001; Type effect (Learning-Related vs DNA Replication-Related): p < 0.0001; Interaction: p = 0.0018. Post-hoc multiple comparisons for H4K12ac levels (Sidak’s multiple comparison test): Learning-Related Genes: adjusted p-value < 0.0001 (****); DNA Replication-Related Genes: adjusted p-value = 0.3293.

Global acetylation of H4K12 is greatly reduced in TIP60-deficient nuclei (Fig. 4A,B). In order to determine at which genes H4K12 was hypoacetylated hippocampal neurons were sorted by FACS and subjected to chromatin immunoprecipitation followed by deep sequencing (ChIP-Seq). PCA showed a clear separation of the genotypes (Fig. 5A). As evident from the heatmap in Fig. 5B the majority of promoter regions with significantly changed H4K12ac binding (adjusted p-value < 0.05 and |fold change| > 1.2) have a reduction of H4K12ac levels in *Tip60* cKOs. We performed GO analysis with these H4K12 hypoacetylated genes and found many of the same categories that had previously been identified for genes with downregulated transcripts (Figs 5C and 4E).

We next compared H4K12ac binding at the promoters of all downregulated genes identified by RNA-Seq (Supplementary Table S3). The heatmap in Fig. 5D clearly shows that the differences between control and *Tip60* cKO with regard to presence of H4K12ac at promoters are highly consistent for the downregulated genes. This consistency is not found for H4K12ac binding within the gene bodies (Fig. 5E). Profile plots show a significant reduction in H4K12ac binding at downregulated genes, especially around the TSS regions both using a stricter (Fig. 5F,G) and a more lenient cut-off (Supplementary Figure S5). H4K12ac levels at upregulated genes did not show any significant change in *Tip60* cKO. Moreover, basal levels of H4K12ac were higher at genes that are downregulated in *Tip60* cKO than at upregulated ones (Fig. 5F,G and Supplementary Figure S5). In line with this observation, we also observed a more substantial decrease in H4K12ac at learning-related genes that were downregulated in *Tip60* cKO than at DNA replication-related genes that were upregulated in these mice (Fig. 5H,I). Again, the basal H4K12ac levels were higher at learning-related genes than at DNA replication-related ones.

In summary, downregulated genes are specifically affected by decreased H4K12ac binding, especially at their TSS, while upregulated genes are not affected. Moreover, downregulated genes exhibit higher basal H4K12ac levels than the upregulated ones, supporting a direct involvement of the acetylation of H4K12 in the control of expression of downregulated genes. This is consistent with the notion that H4K12 acetylation correlates with active transcription^33^.

### Transcriptional changes upon *Tip60* loss resemble those seen in the CK-p25 mouse model for neurodegeneration

**Figure 6:**
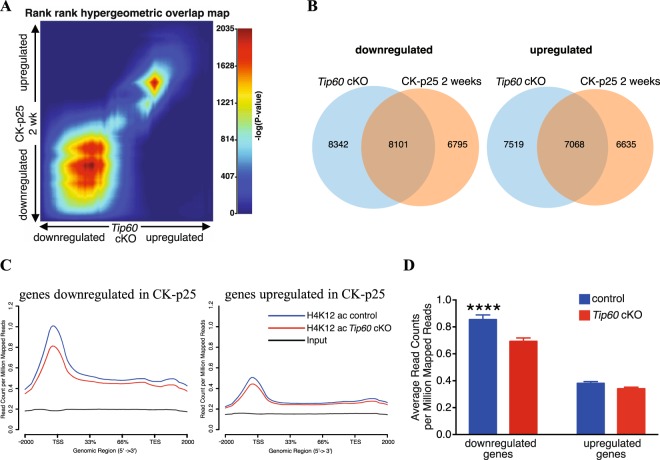
Significant overlap between dysregulated genes and GO categories in CK-p25 and TIP60-deficient mice. (**A**) Rank-rank hypergeometric overlap analysis was used between genes dysregulated in *Tip60* cKO and those in CK-p25 mice induced for 2 weeks (ref.^36^; GSE65159). Each pixel represents a comparison for one gene color-coded for significance (max –log_10_(p-value) = 2035). The most downregulated genes are at the bottom left corner and the most upregulated ones at the upper right corner of the heatmap. (**B**) Venn diagrams representing significantly overlapping down- and upregulated genes in *Tip60* cKO and CK-p25 mice (2 weeks induction) (ref.^36^; GSE65159). In each case, the overall significance of the overlaps was determined by permutation tests in which 1000 permutations were performed yielding a highly significant permutation p-value of < 0.0001. (**C**) H4K12 acetylation levels in *Tip60* cKOs and controls at genes downregulated (left) and upregulated (right) in CK-p25 mice. Genes downregulated in CK-p25 mice have significantly decreased H4K12ac levels around their TSS (+ / −2000 bp) in the *Tip60* cKO, whereas the upregulated ones do not. Moreover, downregulated genes have higher basal levels of H4K12ac compared to upregulated ones. (**D**) Quantification of the profile plots in (C). Genes downregulated in 2-week induced CK-p25 mice (ref.^36^; GSE65159) have significantly decreased H4K12ac levels around their TSS (+ / −2000 bp) in *Tip60* cKO, whereas the upregulated ones do not. Moreover, downregulated genes have higher basal levels of H4K12ac compared to upregulated ones. Two-Way ANOVA: Genotype effect (control vs CK-p25): p < 0.0001; Direction effect (downregulated vs upregulated): p < 0.0001; Interaction: p = 0.0085. Post-hoc multiple comparisons for H4K12ac levels (Sidak’s multiple comparison test): downregulated genes: adjusted p-value < 0.0001 (****); upregulated genes: adjusted p-value = 0.3955.

We showed that loss of *Tip60* already 10 days after gene deletion is associated with decreased neuronal H4K12ac and a downregulation of learning-related genes. Eventually, these changes in the transcriptome are followed by neuronal cell loss. This prompted us to compare the transcriptional changes observed in *Tip60* cKO mice with those of the CK-p25 mouse model of neurodegeneration^34^. Expressing p25 (a proteolytic fragment of p35) under the control of the CaMK2 promoter hyperactivates Cdk5 in excitatory neurons. This leads to significant neuronal and synaptic loss already at week 6 after induction^35^. These neurodegenerative processes are accompanied by neuroinflammation and result in hippocampal atrophy, as is the case in *Tip60* cKO hippocampus. Transcriptomes 10 days after *Tip60* deletion and 2 and 6 weeks after CK-p25 transgene induction (ref.^36^, GEO: GSE65159) were compared. First, we overlapped down- and upregulated genes in both transgenic models (adjusted p-value < 0.05 and |fold change| > 1.2, Supplementary Table S4). For the downregulated genes there was a substantial overlap for both time points (Supplementary Figure S5). For upregulated genes this overlap was less pronounced (Supplementary Figure S5). Next, we applied rank-rank hypergeometric overlap (RRHO) analysis to identify potential concordances between dysregulation in the two models. This approach allows for a threshold free identification of overlaps followed by quantification of significance using multiple testing correction and permutation testing^37^. We identified a significant overlap between down- and upregulated genes in *Tip60* cKO and CK-p25 transgenic mice, at 2 weeks (Fig. 6A,B, Supplementary Tables S5 and S6) and 6 weeks (Supplementary Figure S5, Supplementary Tables S7 and S8). The RRHO maps in Fig. 6A and Supplementary Figure S5 demonstrate that this overlap was far more prominent in downregulated genes than in upregulated ones. When we performed functional annotation analysis for the dysregulated genes common to both models, we found many of the same functional groups that we had already seen upon deletion of *Tip60* in CA1 (Fig. 4E). This included learning and synaptic plasticity-related functions (downregulated) and DNA replication and immune response-related functions (upregulated) (Supplementary Figure S6).

Next, those genes that were significantly dysregulated in CK-p25 mice were analyzed for their H4K12 acetylation status in *Tip60* cKO hippocampus. Downregulated genes showed a significant decrease in H4K12ac binding at the TSS while upregulated ones were not affected both at 2 weeks (Fig. 6C,D) and 6 weeks (Supplementary Figure S6) after induction. The significant overlap between the TIP60-deficient and CK-p25 overexpressing mouse lines corroborates our TIP60 transcriptomics data as well as our histological evidence for a role of TIP60 in neurodegeneration.

### *Tip60* cKO mice exhibit memory impairment and behavioral abnormalities

**Figure 7:**
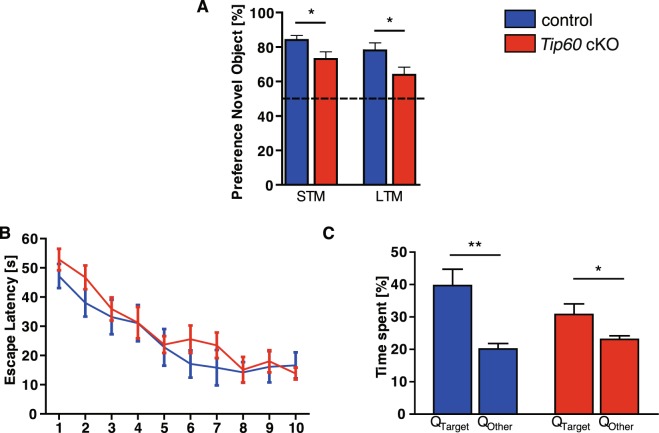
TIP60-deficient mice show modest memory impairment. (**A**) Novel object recognition. *Tip60* cKO mice showed significantly less preference for the novel object than controls when tested 5 min (short-term memory test, STM) and 24 hours (long-term memory test, LTM) after training (*p < 0.05). Chance level is depicted by the dashed line. n = 9–11. (**B**) Morris water maze. Escape latency to find the hidden platform was not significantly different throughout the training days between *Tip60* cKO mice and controls (n = 9–11; repeated-measures two-way ANOVA, F_(1,18)_ = 0.5274, genotype effect p = 0.4771). (**C**) During probe test, both control and TIP60-deficient mice spent significantly more time in the target quadrant (Q_Target_), when compared to the other 3 quadrants (Q_Other_) (**p < 0.01, *p < 0.05). Error bars represent SEM.

In keeping with the findings that KATs are involved in learning and memory, we sought to assess memory function in *Tip60* cKO mice using behavioral analysis. We noticed that *Tip60* cKO mice were “jumpy”, sometimes showed limb clasping (Figure S7) and sporadic seizures. These abnormalities as well as the significant neuronal loss we observed in aging *Tip60* cKO mice prevented reliable behavioral testing beyond two months after *Tip60* deletion. Therefore, we conducted our behavioral experiments between 3 to 6 weeks after *Tip60* deletion. *Tip60* cKO mice performed significantly worse than controls in both the short-term and long-term test of object recognition memory (Fig. 7A; p < 0.05). When tested for spatial memory in the Morris water maze, there was no significant difference between the genotypes regarding the escape latency during the 10 days of training (Fig. 7B; p = 0.4771). *Tip60* cKO mice showed trends for increased escape latency during the learning trials and for decreased time spent in the target quadrant (Fig. 7C; p = 0.0021 controls, p = 0.0374 *Tip60* cKOs). However, these trends did not reach significance. Swim speed was comparable between control and TIP60-deficient animals (Figure S7). No changes were found in contextual fear conditioning as evident by comparable freezing levels between genotypes (Figure S7; p = 0.4186). In the open field test *Tip60* cKOs and controls spent a comparable amount of time in the center of the arena (Figure S7; p = 0.5292). Also the distance travelled (p = 0.1093) and the speed (p = 0.1085) were comparable between genotypes (Figure S7). When tested in the elevated plus maze *Tip60* cKO mice spent significantly more time in the open arms, indicating an anxiolytic effect of TIP60-deficiency (Figure S7, p < 0.01).

## Discussion

The counteracting activities of KATs and KDACs determine the acetylation status of histone and non-histone proteins. A disturbed acetylation homeostasis causes transcriptional dysfunction and is implicated in age-associated learning impairment and in neurodegenerative diseases^4,12,38^. *Tip60* and *Gcn5* are the two most highly expressed KATs in CA1^14^. Little is known about the physiological function of TIP60 in the mammalian brain. We generated a conditional allele to delete *Tip60* in postmitotic excitatory neurons of the adult forebrain. Viable mutant mice made it possible, for the first time, to study the *in vivo* function of TIP60 in the mammalian hippocampus. In the present study we focus in particular on CA1, whose biochemical, molecular and cellular status was examined at defined time points after *Tip60* deletion. Within 10 days, no TIP60 protein was detected in mutant cells and acetylation of *Tip60* target histone H4K12 was reduced by 60% in these cells. ChIP-Seq results show that this reduction was most prominent in the TSS of genes downregulated in *Tip60* cKO. The CA1 transcriptome of mutant mice showed massive changes, indicating major transcriptional dysfunction. The magnitude of misregulation of gene expression in *Tip60* cKO mice exceeds by far all previously studied KAT loss of function mouse models. For example, deletion of *Gcn5* results in much fewer dysregulated genes representing pathways different from those seen in *Tip60* cKOs^14^. Knockouts of other KATs, such as p300 and CBP, also showed comparatively modest changes in transcription^39–42^.

GO analysis suggested that genes downregulated in *Tip60* cKOs mediate synaptic plasticity and learning and upregulated genes drive cell cycle re-entry and neuroinflammation. Several studies suggest that this combination of GO categories is a hallmark of neurodegeneration^11,43,44^. One model that has been extensively characterized in this context is the CK-p25 mouse^34–36^. After 6 weeks of p25 induction these mice show progressive neuronal death in the hippocampus, accompanied by astrogliosis^34,35^. They also show reduced H4K12 acetylation at neuroplasticity genes^12^. When we compared the differentially expressed genes for the *Tip60* cKO model with those of the CK-p25 model of neurodegeneration we found a significant overlap between both the up- and the downregulated genes. When these overlapping genes were subjected to GO categorization, we found many of the same GO categories that had emerged from the GO analysis of the differentially regulated genes of the *Tip60* cKO model. These crosswise comparisons support the existence of a neurodegeneration-related signature in *Tip60* cKOs, already shortly after *Tip60* deletion, and prior to any cellular and morphological changes. We could further show that for the overlapping downregulated genes promoter-associated H4K12 was almost exclusively hypoacetylated, implicating a direct involvement of this histone mark in the observed gene expression signature. Of note, *Hdac2*, which counteracts TIP60-mediated acetylation of histone H4, is upregulated in CK-p25 mice^12^ but unchanged in *Tip60* cKOs and leads to the downregulation of neuroplasticity-related genes via an epigenetic blockade of acetylation of several histones including H4K12ac^12^. This data together with the TIP60 data presented here, strongly supports the idea that the balance between HDAC2 and TIP60 activities mediates H4K12ac-dependent expression of neuronal plasticity-related genes. Shifting this equilibrium between HDAC2 and TIP60 activity, as occurs in Tip60 cKOs, appears to compromise acetylation homeostasis thereby jeopardizing long-term neuronal survival.

Indeed, in *Tip60* cKO mice the transcriptional dysfunction presages a neurodegeneration phenotype. Beginning at 3 weeks after *Tip60* deletion, cleaved Caspase 3 and Fractin signals emerged in medial sections throughout CA1, especially in the *stratum radiatum* and *stratum oriens*. By 3 months, Caspase 3 and Fractin signals had massively spread in a medial to lateral direction. Other studies have previously shown that Caspase 3 activity is present in synaptic and dendritic degenerative processes^25,45,46^. At 9 months, the thickness of *Tip60* cKO CA1 stratae was half from that seen in controls and there was massive neuronal inflammation. No other KAT-deficiency mouse model displays such a neurodegenerative phenotype.

*Tip60* cKO mice exhibited, by 3–6 weeks after gene deletion, and prior to any substantial neuronal loss, a significant impairment in object recognition memory. Other forms of memory were not significantly affected. Interestingly, mouse models with conditional loss of other KATs such as CBP also exhibit impaired object recognition memory, while spatial learning in the Morris water maze was reported to be impaired only in some but not all studies of these mice^8,39,47^. Loss of GCN5 causes a very robust impairment in spatial learning while novel object recognition is rather mildly impaired^14^.

The present study focused on gene classes whose expression is associated with the TIP60-mediated acetylation of H4K12. However, TIP60 also has non-histone targets and can act as a transcriptional co-repressor (reviewed in ref.^21^). Its widespread effects on transcriptional regulation are highlighted by studies in other model organisms. TIP60 is the only essential HAT in yeast^48,49^ and forms a highly interconnected hub of transcriptional regulation^50,51^. Moreover, Lehner *et al*.^50^ identified *Tip60* as one of six genes in *C. elegans* that are highly conserved and modulate diverse signaling pathways. A genome-wide binding study carried out by Ravens *et al*.^52^ suggests that the TIP60 complex functions as a global transcriptional co-activator at the majority of active Pol II promoters and also acts on a subset of enhancers in ES cells. Altogether, these data as well as our own study emphasize that TIP60 has broad control over many cellular processes and does so by affecting transcription. Its loss in excitatory neurons of the brain evokes a disturbance of the acetylation homeostasis, concomitant with the downregulation of neuronal plasticity-related genes, eventually leading to substantial neurodegeneration in the hippocampus.

## Methods

### Animals and treatment

A conditional *Tip60* allele was generated using standard recombineering methods resulting in the targeted allele shown in Fig. 1A. In short, a 15.7 kb fragment of *Tip60* genomic DNA was cloned from a BAC clone (bMQ-331N14, Sanger Institute) into a targeting vector carrying a Pol2-DTA cassette for negative selection. The first loxP site was inserted 637 bp upstream of the *Tip60* ATG. The second loxP site together with a FRT-flanked PGK-neo cassette was inserted 3,065 bp downstream of the ATG. The 5′ end of the homologous arm of the targeting vector was 4.5 kb and the 3′ end of the homologous arm was 7.6 kb. ES cell targeting and generation of *Tip60*^*fl/+*^ founder mice was commissioned to PolyGene Transgenics (Switzerland). The FRT-flanked PGK-neo selection cassette was removed by crossing mice with a Flippase expressing deleter line^53^. Primers for genotyping were 5′-TCAGAAGATGCACCTTCTGCTGG-3′ (I), 5′-GGAAGGTTCAAAATTCCAGTAGGC-3′ (II), and 5′-TGCTTCCGCTTCCTGAATGCTG-3′ (III) and product sizes were 331 bp for the wild type, 429 bp for the floxed, and 487 bp for the KO allele. The tamoxifen-inducible *CaMKCreER*^*T2*^ mouse line [Tg(Camk2a-Cre/ERT2)2/Gsc, EM:02125; ref.^24^] was used as a driver. Mice hemizygous for the *CaMKCreER*^*T2*^ transgene (under a C57BL/6 N background) were crossed to *Tip60*^*f/f*^ mice in a C57BL/6 N background to obtain *Tip60* cKO experimental animals and controls. Mice were housed under standard conditions with food and water *ad libitum*. Littermates harboring floxed *Tip60* alleles but negative for the CRE-driver served as controls. Both *Tip60* cKO and control animals of either sex were tamoxifen-injected between 8 and 10 weeks of age. Tamoxifen (T5648, Sigma) was dissolved in corn oil (C8267, Sigma) on a rotating wheel at 37 °C for 6 hours. The stock concentration was 20 mg/ml and mice received 100 µl twice daily via i.p. injections for five consecutive days. All experiments were approved by the Lower Saxony State Office for Consumer Protection and Food Safety (LAVES) and performed in accordance with the German Law of Animal Welfare.

### Antibody production

A custom TIP60-specific antibody (TIP60P4) was generated using a peptide comprising the last 15 amino acids of the TIP60 C-terminus (Immunoglobe Antikörpertechnik GmbH, Himmelstadt, Germany). A tandem purification strategy was employed, which included a first purification using the immunization peptide, CLHFTPKDWSKRGKW, amidated at the C-terminal end. The flow-through of this first purification was then purified using a shorter version of the immunization peptide, SKRGKW. The tandem-purified antibody was specific and sufficiently sensitive to detect endogenous TIP60 in cryosections of mouse brain.

### Immunohistochemistry (IHC) and deletion efficiency analysis

Mice were sacrificed by cervical dislocation and freshly dissected brains were frozen in OCT and cryosectioned sagittally at 10 µm. Sections were transferred to Superfrost slides and stored at −20 °C until further use. Sections were thawed for 10 min at room temperature (RT) and then fixed in 4% PFA for 20 min. Next, they were washed 3 times in 1x PBS, incubated in 0.25% Triton in 1x PBS for 15 min and then in blocking reagent (5% BSA, 1% normal goat serum in 1x PBS) for 1 hour at RT, followed by a single wash in 1x PBS for 10 min. Primary antibody incubation was performed in blocking reagent at 4 °C overnight. Antibodies used were Synaptophysin (101004, 1:500), IBA1 (234003, 1:1000), MAP2 (188004, 1:500), from Synaptic Systems, NeuN (MAB377, 1:100), Fractin (AB3150, 1:500), Acetyl-Histone H4K12 (17-10121, 1:1000) from Millipore, GFAP (4674, 1:500) from abcam, H4ac (39967, 1:1000) from Active Motif, cleaved Caspase 3 (9661, 1:600) from Cell Signaling, TIP60P4 (custom-made, 1:25, see above) and TIP60 C-7 (sc-166323, 1:100) from Santa Cruz. The next day, sections were washed 3 times in 1x PBS for 10 min and incubated with Alexa-conjugated secondary antibodies (Invitrogen) diluted 1:500 in blocking reagent for 1 hour at RT. Sections were washed again 3 times in 1x PBS for 10 min and coverslipped in Vectashield Antifade Mounting Medium with DAPI (Vector) or ProLong Gold Antifade Mountant with DAPI (Invitrogen). Images were captured using an inverted microscope (Leica DMI6000B). For deletion efficiency analysis, composite images of the hippocampus of *Tip60* cKO mice were used. The total number of cells in the principal cell layer of each subregion was determined by quantifying the number of DAPI-stained nuclei using Cell Profiler^54^. For the CA2 region the number of DAPI-stained nuclei was counted manually. Residual TIP60 wild-type cells were detected using TIP60P4 antibody, quantified manually and normalized to the total cell number in each principal cell layer.

### Quantification of histone acetylation

Cell Profiler^54^ or custom-made software (available at GitHub: https://github.com/epicodic/cell_label_tool) was used to quantify the mean gray value. The median pixel intensity across the image was subtracted in order to correct for background.

### Behavior

Only male mice were used for behavioral testing. Tests for Open field and Morris water maze were completed within 3–4 weeks after tamoxifen injections. Novel Object recognition was performed at 4–5 weeks and contextual fear conditioning as well as the elevated plus maze at 5–6 weeks after injections.

#### Open field and novel object recognition

A grey plastic box served as the testing arena for both open field and novel object recognition tests. For the open field test the relative time spent in the center of the testing arena was quantified. For novel object recognition, the mice were exposed to the testing arena for 5 min on the first day. On the next day they were habituated to two white boxes for 5 min. Short-term memory (STM) testing took place on the third day. The mice were first introduced to two black cubes, which they were left to explore for 5 min. The following 5 min they spent in their home cages and were then reintroduced to the testing arena, where one black cube was now exchanged for a stone. For long-term memory (LTM) testing, the mice were introduced to one black cube and a blue screw cap 24 hours later. As a readout of memory performance the relative exploration time of the novel object, i.e., stone (STM) or blue screw cap (LTM), was used.

#### Contextual fear conditioning

To test associative learning mice were placed into a box. After 3 min of exploration, an electrical foot shock (0.7 mA, 2 s) was delivered through the grid floor and the mice removed from the box after another 30 s. 24 hours later, the mice were reintroduced to the box and freezing was measured for 3 min.

#### Elevated plus maze

Mice were placed into the center of the plus maze, which consisted of two arms with walls (“closed”) and two arms without any boundaries (“open”), and were allowed to explore for 5 min. The relative time spent in the open arms was quantified.

#### Morris water maze

A platform was hidden in a circular pool filled with opaque water. The test was performed on 10 consecutive days consisting of 4 training sessions each. At each session, mice were introduced into the pool and left to search for the platform for 60 s. If unsuccessful within the time limit, they were gently guided to the platform. Probe test (without the platform) was carried out 24 hours after the last training session.

### Microdissection and RNA isolation followed by deep sequencing

Microdissection of the dorsal CA1 region (modified from ref.^55^) was performed in ice-cold 1x PBS under a binocular (Leica). Samples from both hemispheres were pooled and transferred into RNALater (Ambion). RNA isolation and deep sequencing were performed at the Transcriptome Analysis Lab (TAL, Göttingen, Germany). Total RNA was isolated using TRIzol (Invitrogen) and nucleic acid quantity, quality and purity were determined using a NanoDrop spectrophotometer and a 2100 Bioanalyzer. Library preparation (starting from 1 μg of total RNA), sequencing and raw data analysis was performed as described previously^56^. Reads were aligned to mouse genome *Mus musculus* mm10 and counted using FeaturesCount (http://bioinf.wehi.edu.au/featureCounts/) as described previously^57^. PCA plots were created and differential expression, including the p-value adjustment, was performed using DESeq. 2 package of Bioconductor^58^. Raw data (fastq files) for gene expression in CK-p25 mice was obtained from GSE65159^36^ and subjected to the same procedures.

### Transcription factor enrichment analysis

Transcription factor enrichment analysis was performed using ToppGene (https://toppgene.cchmc.org/enrichment.jsp). From the output the section “11. Transcription Factor Binding Site” was selected.

### *In situ* hybridization (ISH)

ISH was performed as described previously^59^. Probes used are listed in Supplementary Methods.

### Real-time qPCR

RNA was reverse transcribed using the Quantitect Reverse Transcription Kit (Qiagen). qPCR using SYBR green (Biorad) was performed for 41 cycles (10 s at 95 °C, 25 s at 60 °C, 20 s at 72 °C) using the CFX96 Real-Time PCR Detection System (Biorad). Each sample was used in duplicate and *Gapdh* was taken for normalization^60^. Primers are listed in Supplementary Methods.

### Chromatin immunoprecipitation (ChIP) from FACS-sorted nuclei

ChIP-Sequencing from neuronal nuclei sorted by FACS was performed as described previously^61^ with minor modifications. Whole hippocampal tissue was used. Input chromatin (50 ng) was isolated from every sample and was later pooled. Library preparation was performed using NEBNext Ultra DNA Library Prep Kit for Illumina (NEB). Libraries were measured in Qubit and Agilent 2100 Bioanalyzer.

Base calling, fastq conversion and quality control were performed as previously described^57,61,62^. The reads were mapped to mouse reference genome (Mus musculus mm10) with STAR aligner v2.3.010. PCR duplicates were removed from each BAM file using rmdup function of samtools^63^. Replicates from the same group were merged into a single BAM file using the merge function of samtools^63^. Profile plots of H4K12ac were created using the merged BAM files from immunoprecipitated samples and input with NGSPlot with non-default parameters^64^. For differential binding analysis we first performed peak calling using MACS213 with q < 0.1. Then the differential binding analysis was performed with the BAM files and peaks for each sample using DiffBind package of Bioconductor with an in-built DESEQ. 2 option^65^.

### Functional enrichment analysis

Enrichment of functional GO categories was performed using topGO package of Bioconductor, using the weighted analysis option in order to avoid documenting broad and redundant categories (Alexa and Rahnenfuhrer, 2016). GO categories with a weighted p-value below 0.001 were considered significant.

### Rank-Rank hypergeometric overlap

The RRHO package of Bioconductor was used to detect the extent and significance of overlap between dysregulation in Tip60 cKO and CK-p25 mice^37^. One-sided enrichment tests were implemented with default step size and p-values were corrected for multiple testing using Benjamini-Yekutieli method. Moreover, the significance of the overlaps was additionally tested against 1000 permutations.

### Statistical analysis

Student’s t test was used unless indicated otherwise. Error bars in graphs represent SEM.

## Supplementary information

Supplementary Information

Supplementary Table S1

Supplementary Table S2

Supplementary Table S3

Supplementary Table S4

Supplementary Table S5

Supplementary Table S6

Supplementary Table S7

Supplementary Table S8

## Data Availability

The datasets generated and analyzed during the current study are available from the NCBI Gene Expression Omnibus (GEO) repository: GSE139298 (RNA-Seq) and GSE138830 (ChIP-Seq). Availability of custom polyclonal TIP60P4 antibody is restricted due to limited amounts produced.
