# Supplementary material for: TIP60/KAT5 is required for neuronal viability in hippocampal CA1
Source: Sci Rep. 2019 Nov 7;9:16173. doi: 10.1038/s41598-019-50927-1 (PMC6838100; doi:10.1038/s41598-019-50927-1)
Supplement: Supplementary file 1 — Supplementary Information [file 41598_2019_50927_MOESM1_ESM.pdf]

TIP60/KAT5 is required for neuronal viability in hippocampal CA1

Inga Urban[1], Cemil Kerimoglu[2], M. Sadman Sakib[2], Haifang Wang[3], Eva Benito[2,†], Christina Thaller[1], Xunlei Zhou[1,††], Jun Yan[3,4], André Fischer[2,5,*], Gregor Eichele[1,6,*]

[1]Genes and Behavior Department, Max Planck Institute for Biophysical Chemistry, 37077 Göttingen, Germany
[2]Department of Epigenetics and Systems Medicine in Neurodegenerative Diseases, German Center for Neurodegenerative Diseases (DZNE) Göttingen, 37075 Göttingen, Germany
[3]Institute of Neuroscience, State Key Laboratory of Neuroscience, CAS Center for Excellence in Brain Science and Intelligence Technology, Shanghai Institutes for Biological Sciences, Chinese Academy of Sciences, Shanghai 200031, China
[4]University of Chinese Academy of Sciences, Beijing, China
[5]Department for Psychiatry and Psychotherapy, University Medical Center Göttingen, 37075 Göttingen, Germany
[†]Current address: European Molecular Biology Organization (EMBO), 69117 Heidelberg, Germany
[††]Current address: Institute of Anatomy and Cell Biology, University of Heidelberg, 69120 Heidelberg, Germany
[6]Lead contact
*Correspondence should be addressed to
Gregor Eichele, Max Planck Institute for Biophysical Chemistry, Am Faßberg 11, 37077 Göttingen, Germany; Tel: +49 551 201-2701; E-Mail: Gregor.Eichele@mpibpc.mpg.de
and
André Fischer, Department of Epigenetics and Systems Medicine in Neurodegenerative Diseases, German Center for Neurodegenerative Diseases (DZNE) Göttingen, Von-Siebold-Str. 3a, 37075 Göttingen, Germany; Tel: +49 551 39-61211; E-Mail: andre.fischer@dzne.de

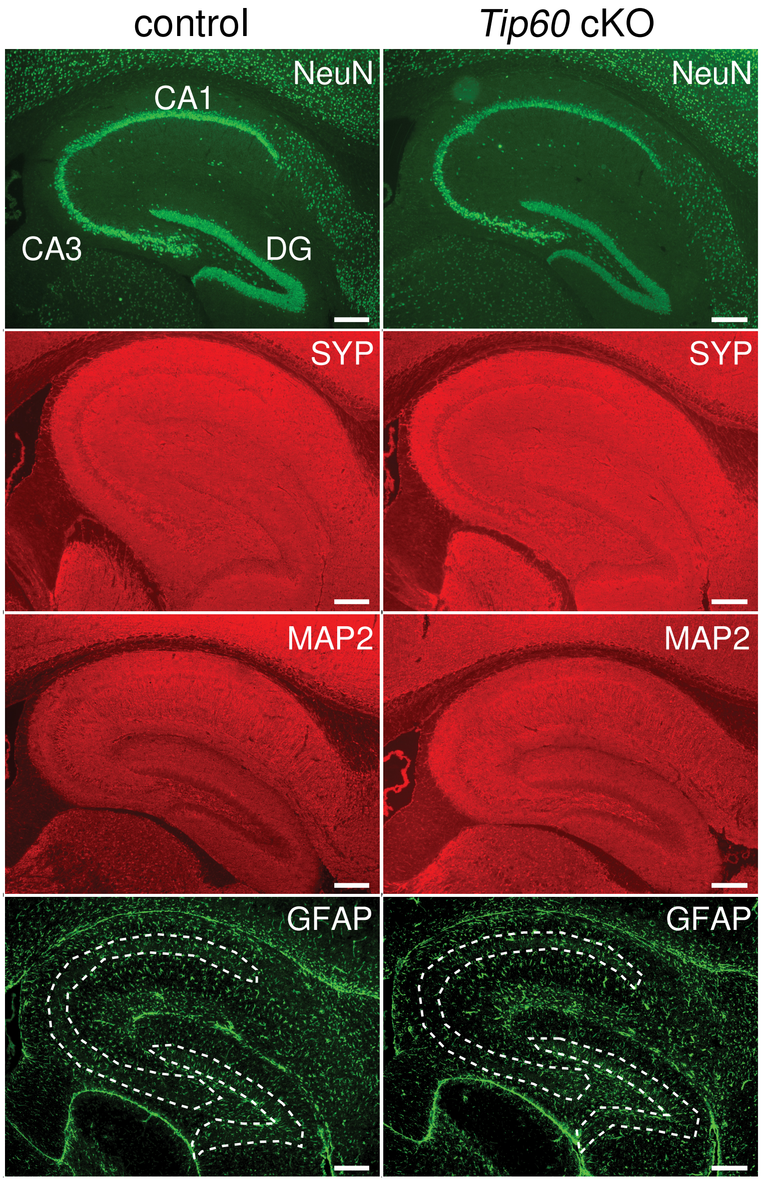

Figure S1: Gross morphology is unaltered in *Tip60* cKO mice at day 10.
Expression of Neuronal Nuclei (NeuN), Synaptophysin (SYP), Microtubule-associated protein 2 (MAP2) and Glial fibrillary acidic protein (GFAP) in control and *Tip60* cKO hippocampus 10 days after tamoxifen injections. Scale bars: 250 µm.

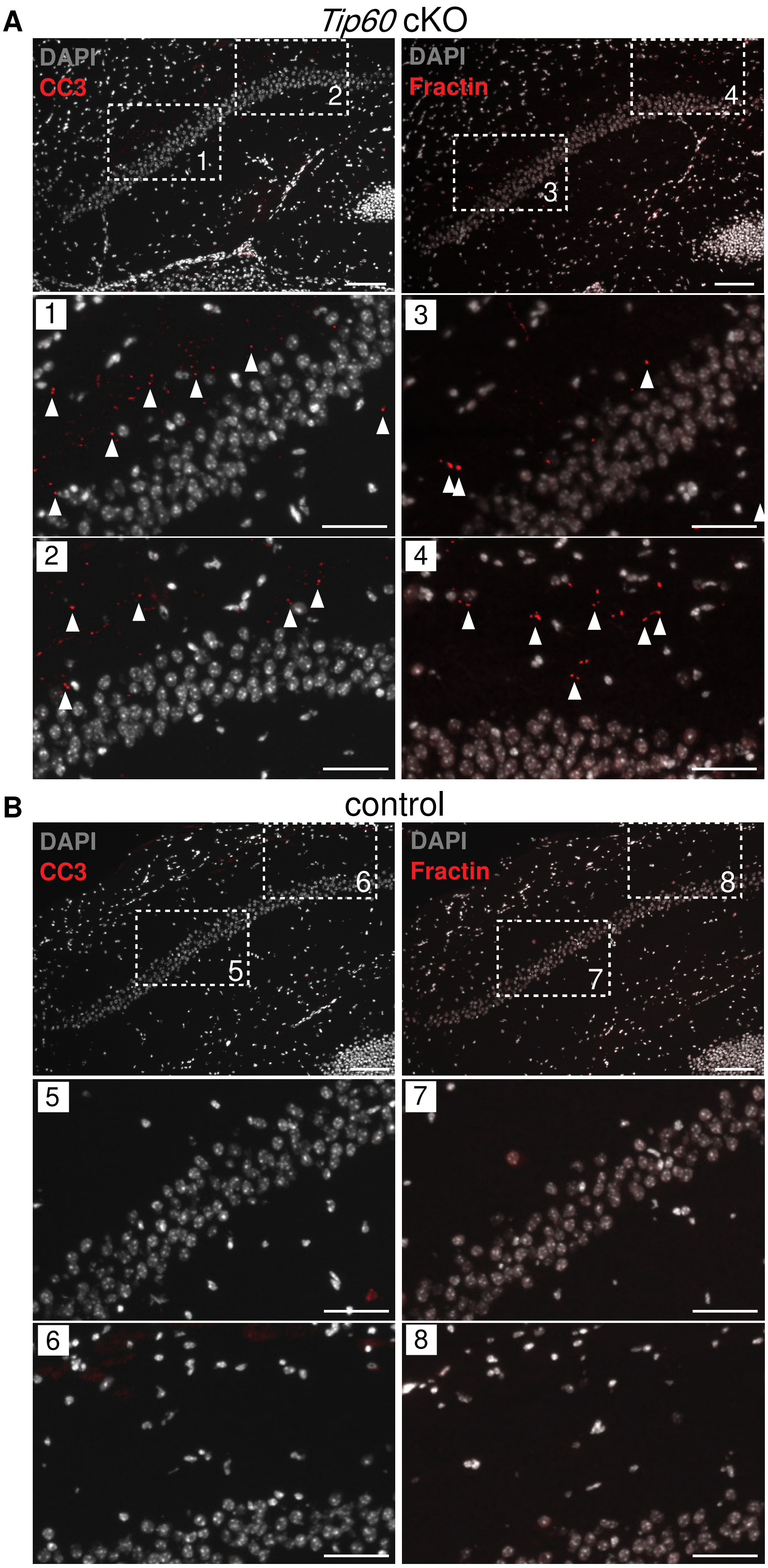
A
Tip60 cKO
DAPI
CC3
1
2
DAPI
Fractin
3
4
1
3
2
4
B
control
DAPI
CC3
5
6
DAPI
Fractin
7
8
5
7
6
8

Figure S2: Punctate cleaved Caspase 3 and Fractin signal in *Tip60* cKOs at 3 weeks.
(A) Punctate cleaved Caspase 3 (CC3) and Fractin signals (arrowheads) are detectable in the most medial sections (~0.475 mm lateral, Paxinos and Franklin, 2001) of the *Tip60* cKO CA1 region at 3 weeks after tamoxifen injections.
(B) Such signals are absent in controls. Scale bars: 100 µm (A, B, top panels), 50 µm (A, B, middle and bottom panels).

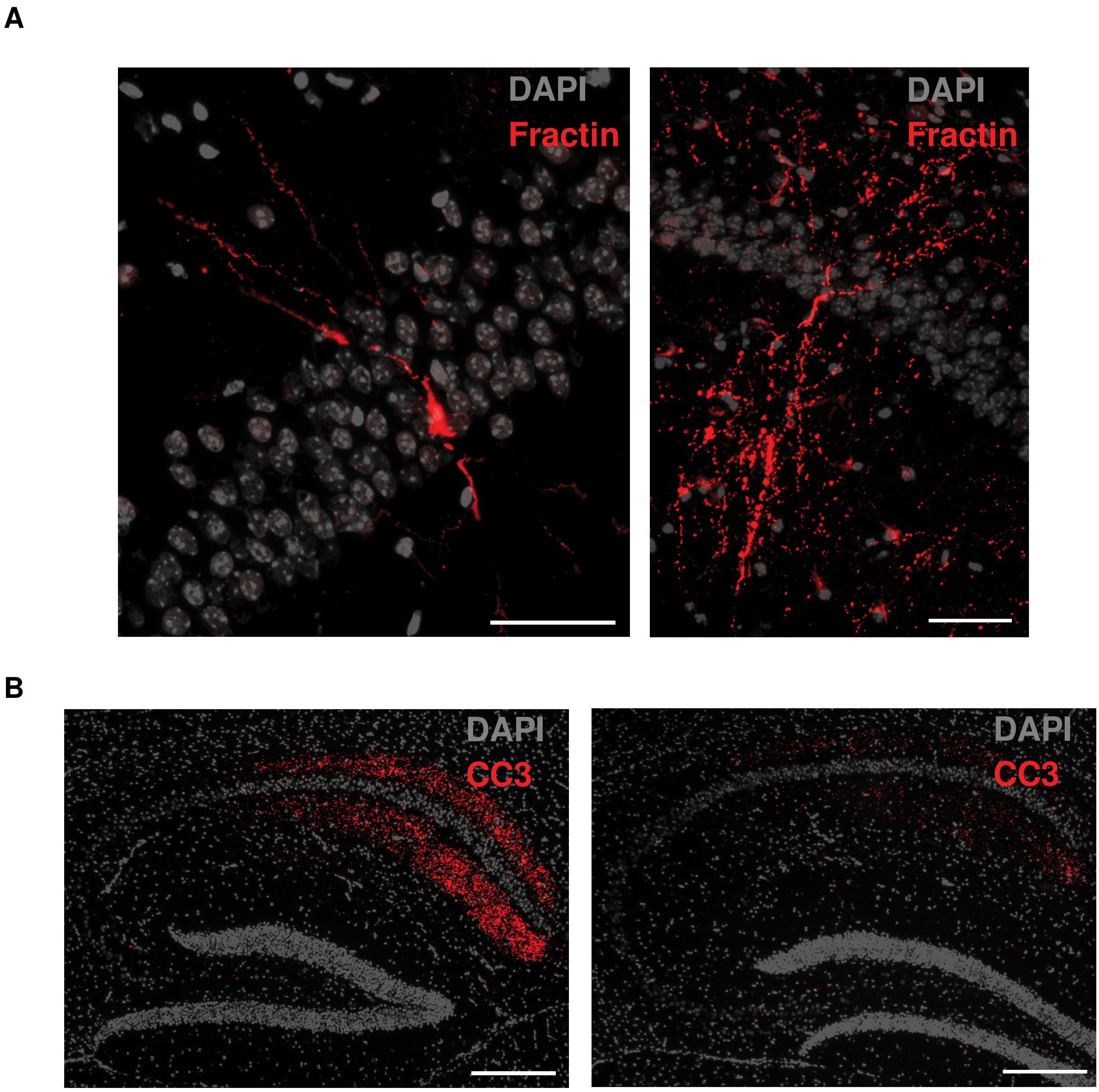

Figure S3: Fractin-positive neurons and medial-to-lateral spreading of cleaved caspase 3 signal.
(A) Fractin-positive neurons in the CA1 region of a *Tip60* cKO animal at 3 weeks (left) and 2 months (right) after tamoxifen injections.
(B) Activation of Caspase 3 (CC3) in the TIP60-deficient hippocampus is restricted to CA1 and progressively spreads more laterally with time. Images of two representative sagittal sections from the same *Tip60* cKO brain at 5 months are shown. Note that CC3 signal is more intense in the more medial section (left) compared to the more lateral section (right) and is restricted to the CA1. Sectioning planes were ~1.4 mm (left) and ~2.2 mm (right) lateral (Paxinos and Franklin, 2001). Scale bars: 50 µm (A), 250 µm (B).

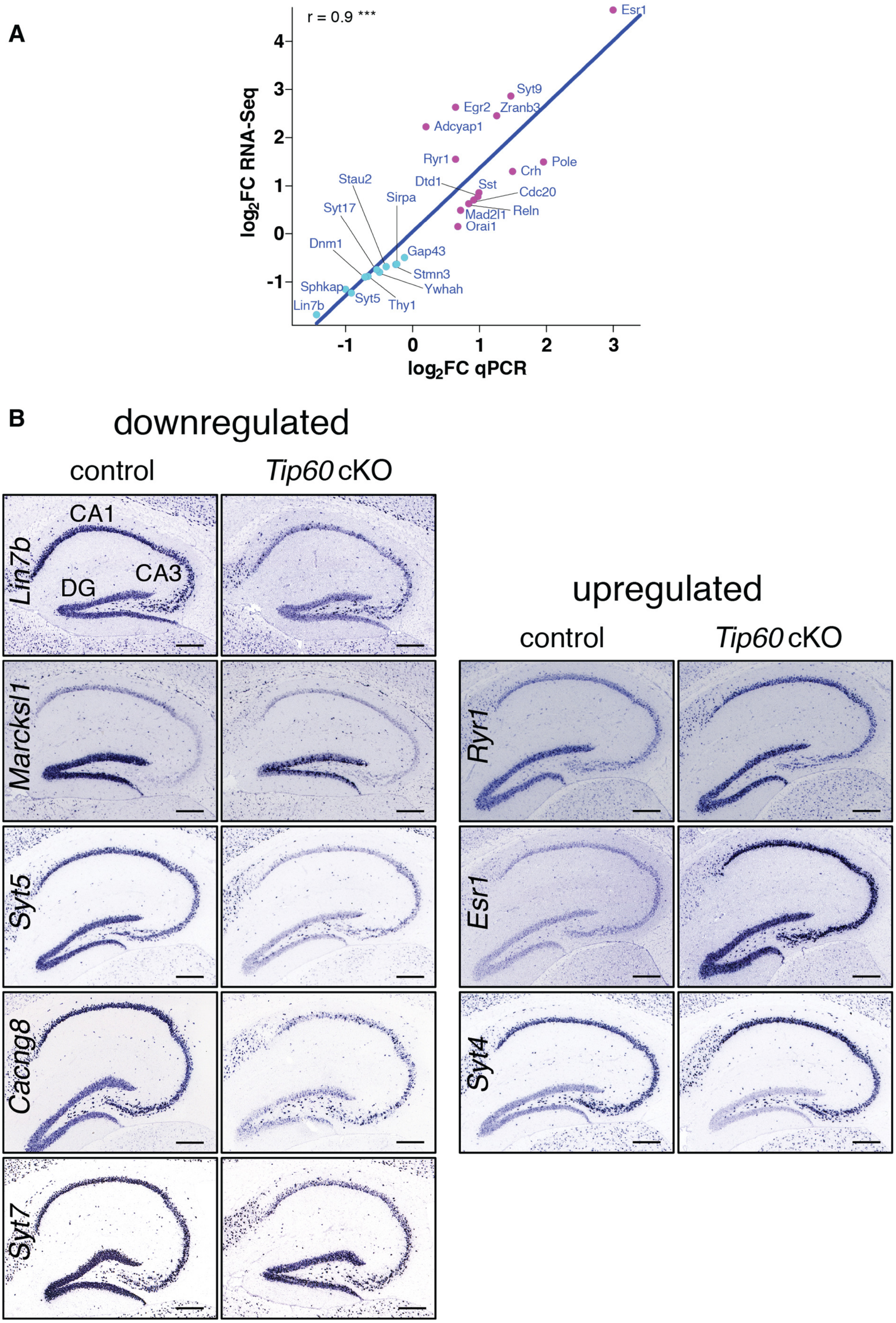
A
r = 0.9 ***
log2FC RNA-Seq
log2FC qPCR
Esr1
Syt9
Egr2
Zranb3
Adcyap1
Ryr1
Pole
Crh
Dtd1
Sst
Cdc20
Mad2l1
Reln
Orai1
Stau2
Sirpa
Syt17
Dnm1
Gap43
Stmn3
Ywhah
Sphkap
Syt5
Thy1
Lin7b
B
downregulated
control
Tip60 cKO
CA1
CA3
DG
Lin7b
Marcksl1
Syt5
Cacng8
Syt7
upregulated
control
Tip60 cKO
Ryr1
Esr1
Syt4

Figure S4: Validation of selected genes significantly changed in RNA-Seq analysis.

(A) Validation of selected, differentially expressed genes by qPCR. Comparison of the log_2-transformed fold changes (log_2FC) of RNA-Seq and qPCR shows strong correlation. Data points for downregulated genes are in cyan and for upregulated genes in magenta. The same samples as for RNA-Seq analysis were used (n = 6 per genotype).

(B) Spatial distribution of selected, differentially expressed transcripts in the hippocampus of control and *Tip60* cKO mice analyzed by ISH. Representative images of hippocampal expression of selected genes dysregulated in *Tip60* cKO mice show that for most transcripts differential expression is not restricted to CA1 but covers all hippocampal subregions (cf. expression of *Lin7b*, *Marcksl1*, *Syt5*, *Cacng8* and *Esr1*). Note that for *Marcksl1* downregulation in DG is even stronger than in CA1 while for *Syt7* it is weaker. Changes of *Syt4* expression in *Tip60* cKO mice depend on the subregion: transcript levels are upregulated in CA1 but downregulated in DG. Cryosections of animals at 10 days after tamoxifen injections were used. n = 4 (*Lin7b*, *Ryr1*, *Esr1*, *Syt4*), n = 3 (*Marcksl1*, *Syt5*), n = 1 (*Cacng8*, *Syt7*) per genotype, 4 sections per animal. Scale bars: 250 µm.

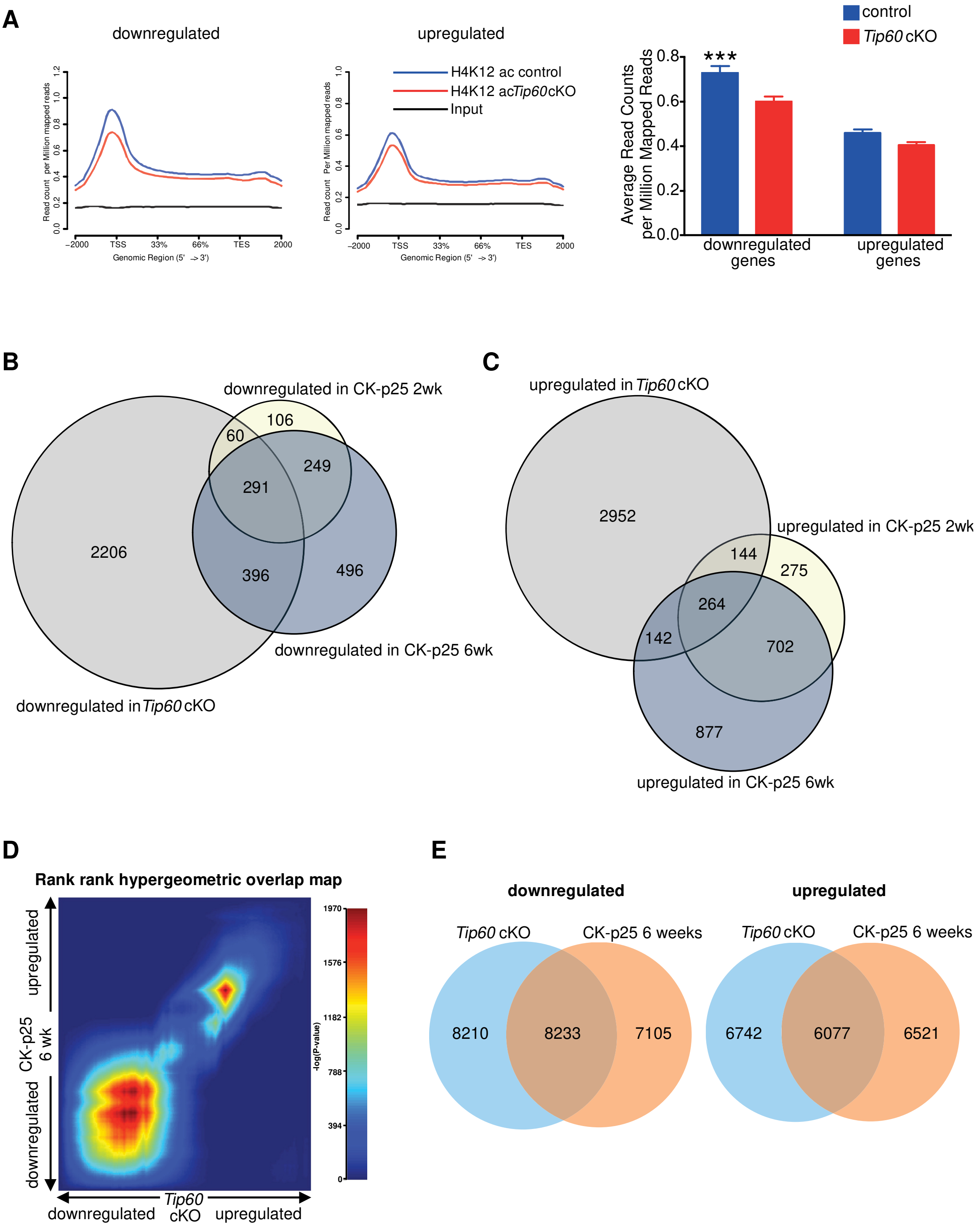
A
downregulated
upregulated
H4K12 ac control
H4K12 acTip60cKO
Input
Read count per Million mapped reads
Genomic Region (5' -> 3')
−2000
TSS
33%
66%
TES
2000
control
Tip60 cKO
Average Read Counts per Million Mapped Reads
***
0.0
0.2
0.4
0.6
0.8
downregulated genes
upregulated genes
B
downregulated in CK-p25 2wk
106
60
249
291
2206
396
496
downregulated in CK-p25 6wk
downregulated in Tip60 cKO
C
upregulated in Tip60 cKO
2952
upregulated in CK-p25 2wk
144
275
264
142
702
877
upregulated in CK-p25 6wk
D
Rank rank hypergeometric overlap map
1970
1576
1182
788
394
0
-log(P-value)
upregulated
CK-p25 6 wk
downregulated
downregulated
Tip60 cKO
upregulated
E
downregulated
upregulated
Tip60 cKO
CK-p25 6 weeks
8210
8233
7105
Tip60 cKO
CK-p25 6 weeks
6742
6077
6521

Figure S5: H4K12ac levels at dysregulated genes in *Tip60* cKOs and overlap of dysregulated genes with the CK-p25 mouse model.

(A) H4K12 acetylation levels at genes downregulated (left) and upregulated (middle) in *Tip60* cKO mice with p-adjusted < 0.05 and |fold change| > 1.2. Genes downregulated in *Tip60* cKO mice have significantly decreased H4K12ac levels around their TSS (+/- 2000 bp), whereas the upregulated ones do not (right). Moreover, downregulated genes have higher basal levels of H4K12ac compared to upregulated ones. Two-Way ANOVA: Genotype effect (control vs *Tip60* cKO): $p < 0.0001$; Direction effect (downregulated vs upregulated): $p < 0.0001$; Interaction: $p = 0.1019$. Post-hoc multiple comparisons for H4K12ac levels (Sidak's multiple comparison test): downregulated genes: adjusted p-value < 0.001 (***); upregulated genes: adjusted p-value = 0.1619.

(B) The overlap between genes downregulated in *Tip60* cKO and those in CK-p25 mice (2 and 6 weeks of induction) (ref[36]; GSE65159).

(C) The overlap between genes upregulated in *Tip60* cKO and those in CK-p25 mice (2 and 6 weeks of induction) (ref[36]; GSE65159).

(D) Rank-rank hypergeometric overlap analysis was used between genes dysregulated in *Tip60* cKO and those in CK-p25 mice induced for 6 weeks (ref[36]; GSE65159). Each pixel represents comparison for one gene color-coded for significance (max –log10(p-value) = 1970). The most downregulated genes are at the bottom left corner and the most upregulated ones at the upper right corner of the heatmap.

(E) Venn diagrams representing significantly overlapping down- and upregulated genes in *Tip60* cKO and CK-p25 mice (6 weeks induction) (ref[36]; GSE65159). In each case, the overall significance of the overlaps was determined by permutation tests in which 1000 permutations were performed yielding a highly significant permutation p-value of < 0.0001.

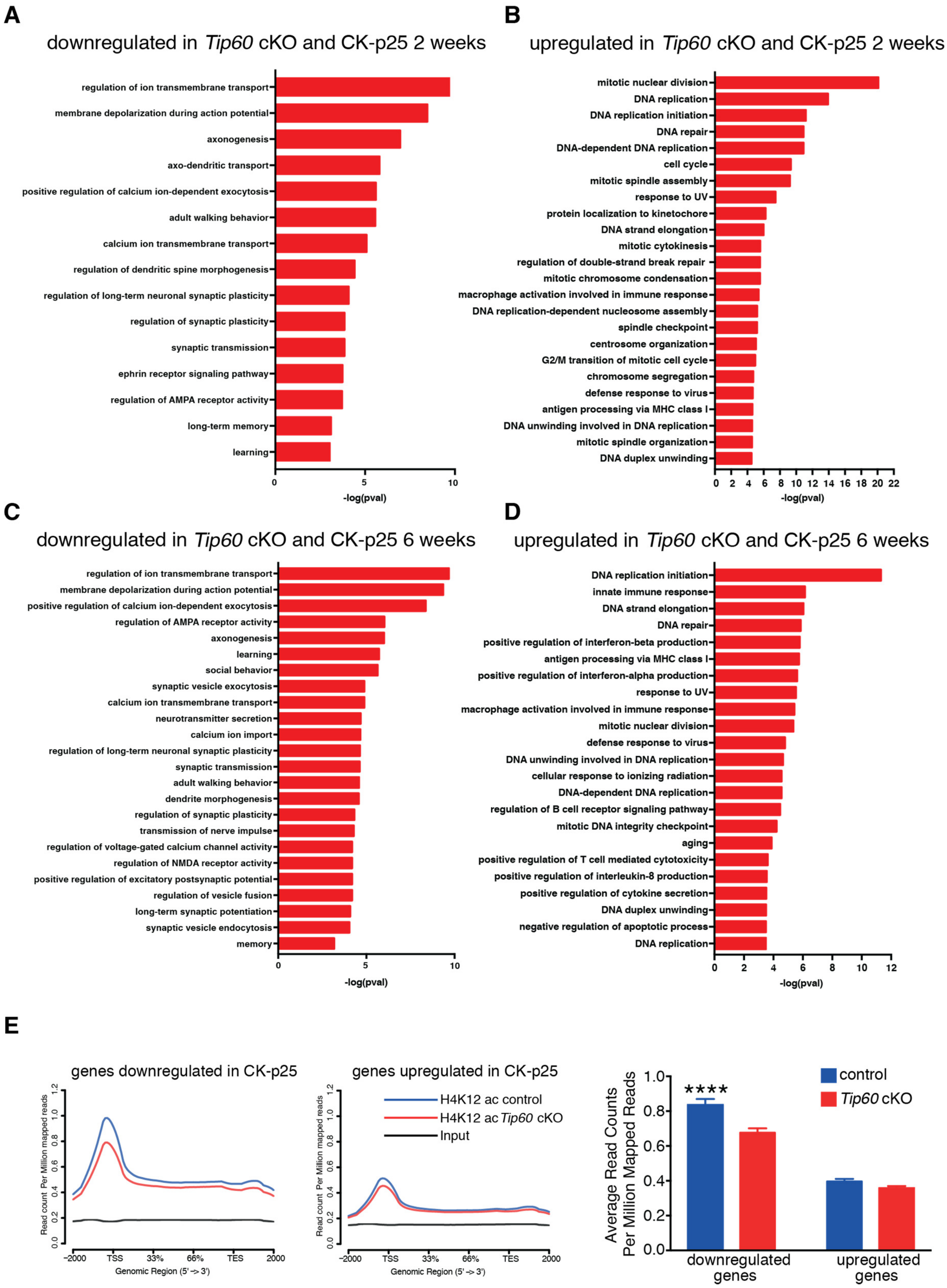

A
downregulated in Tip60 cKO and CK-p25 2 weeks
regulation of ion transmembrane transport
membrane depolarization during action potential
axonogenesis
axo-dendritic transport
positive regulation of calcium ion-dependent exocytosis
adult walking behavior
calcium ion transmembrane transport
regulation of dendritic spine morphogenesis
regulation of long-term neuronal synaptic plasticity
regulation of synaptic plasticity
synaptic transmission
ephrin receptor signaling pathway
regulation of AMPA receptor activity
long-term memory
learning
0
5
10
-log(pval)
B
upregulated in Tip60 cKO and CK-p25 2 weeks
mitotic nuclear division
DNA replication
DNA replication initiation
DNA repair
DNA-dependent DNA replication
cell cycle
mitotic spindle assembly
response to UV
protein localization to kinetochore
DNA strand elongation
mitotic cytokinesis
regulation of double-strand break repair
mitotic chromosome condensation
macrophage activation involved in immune response
DNA replication-dependent nucleosome assembly
spindle checkpoint
centrosome organization
G2/M transition of mitotic cell cycle
chromosome segregation
defense response to virus
antigen processing via MHC class I
DNA unwinding involved in DNA replication
mitotic spindle organization
DNA duplex unwinding
0 2 4 6 8 10 12 14 16 18 20 22
-log(pval)
C
downregulated in Tip60 cKO and CK-p25 6 weeks
regulation of ion transmembrane transport
membrane depolarization during action potential
positive regulation of calcium ion-dependent exocytosis
regulation of AMPA receptor activity
axonogenesis
learning
social behavior
synaptic vesicle exocytosis
calcium ion transmembrane transport
neurotransmitter secretion
calcium ion import
regulation of long-term neuronal synaptic plasticity
synaptic transmission
adult walking behavior
dendrite morphogenesis
regulation of synaptic plasticity
transmission of nerve impulse
regulation of voltage-gated calcium channel activity
regulation of NMDA receptor activity
positive regulation of excitatory postsynaptic potential
regulation of vesicle fusion
long-term synaptic potentiation
synaptic vesicle endocytosis
memory
0
5
10
-log(pval)
D
upregulated in Tip60 cKO and CK-p25 6 weeks
DNA replication initiation
innate immune response
DNA strand elongation
DNA repair
positive regulation of interferon-beta production
antigen processing via MHC class I
positive regulation of interferon-alpha production
response to UV
macrophage activation involved in immune response
mitotic nuclear division
defense response to virus
DNA unwinding involved in DNA replication
cellular response to ionizing radiation
DNA-dependent DNA replication
regulation of B cell receptor signaling pathway
mitotic DNA integrity checkpoint
aging
positive regulation of T cell mediated cytotoxicity
positive regulation of interleukin-8 production
positive regulation of cytokine secretion
DNA duplex unwinding
negative regulation of apoptotic process
DNA replication
0 2 4 6 8 10 12
-log(pval)
E
genes downregulated in CK-p25
Read count Per Million mapped reads
0.0 0.2 0.4 0.6 0.8 1.0 1.2
−2000 TSS 33% 66% TES 2000
Genomic Region (5' -> 3')
genes upregulated in CK-p25
H4K12 ac control
H4K12 ac Tip60 cKO
Input
Read count Per Million mapped reads
0.0 0.2 0.4 0.6 0.8 1.0 1.2
−2000 TSS 33% 66% TES 2000
Genomic Region (5' -> 3')
Average Read Counts Per Million Mapped Reads
0.0 0.2 0.4 0.6 0.8 1.0
****
control
Tip60 cKO
downregulated genes
upregulated genes

Figure S6: GO categories of overlapping genes between *Tip60* cKOs and CK-p25 mice and H4K12ac levels in *Tip60* cKOs at genes dysegulated in CK-p25 mice.

(A, B) GO categories of overlapping downregulated (A) and upregulated (B) genes between *Tip60* cKO mice at 10 days and CK-p25 mice at 2 weeks.

(C, D) GO categories of overlapping downregulated (C) and upregulated (D) genes between *Tip6*0 cKO mice at 10 days and CK-p25 mice at 6 weeks. 2 weeks after p25 induction represent an early and 6 weeks a late stage of neurodegeneration in CK-p25 mice (ref[36]).

(E) H4K12 acetylation levels in *Tip60* cKOs and controls at genes downregulated (left) and upregulated (middle) in CK-p25 mice 6 weeks after induction. Genes downregulated in CK-p25 mice have significantly decreased H4K12ac levels around their TS (+/- 2000 bp), whereas upregulated ones do not (right). Moreover, downregulated genes have higher basal levels of H4K12ac than upregulated ones. Two-Way ANOVA: Genotype effect (control vs *Tip60* cKO): $p < 0.0001$; Direction effect (downregulated vs upregulated): $p < 0.0001$; Interaction: $p = 0.0105$. Post-hoc multiple comparisons for H4K12ac levels (Sidak's multiple comparison test): downregulated genes: adjusted p-value < 0.0001 (****); upregulated genes: adjusted p-value = 0.4190.

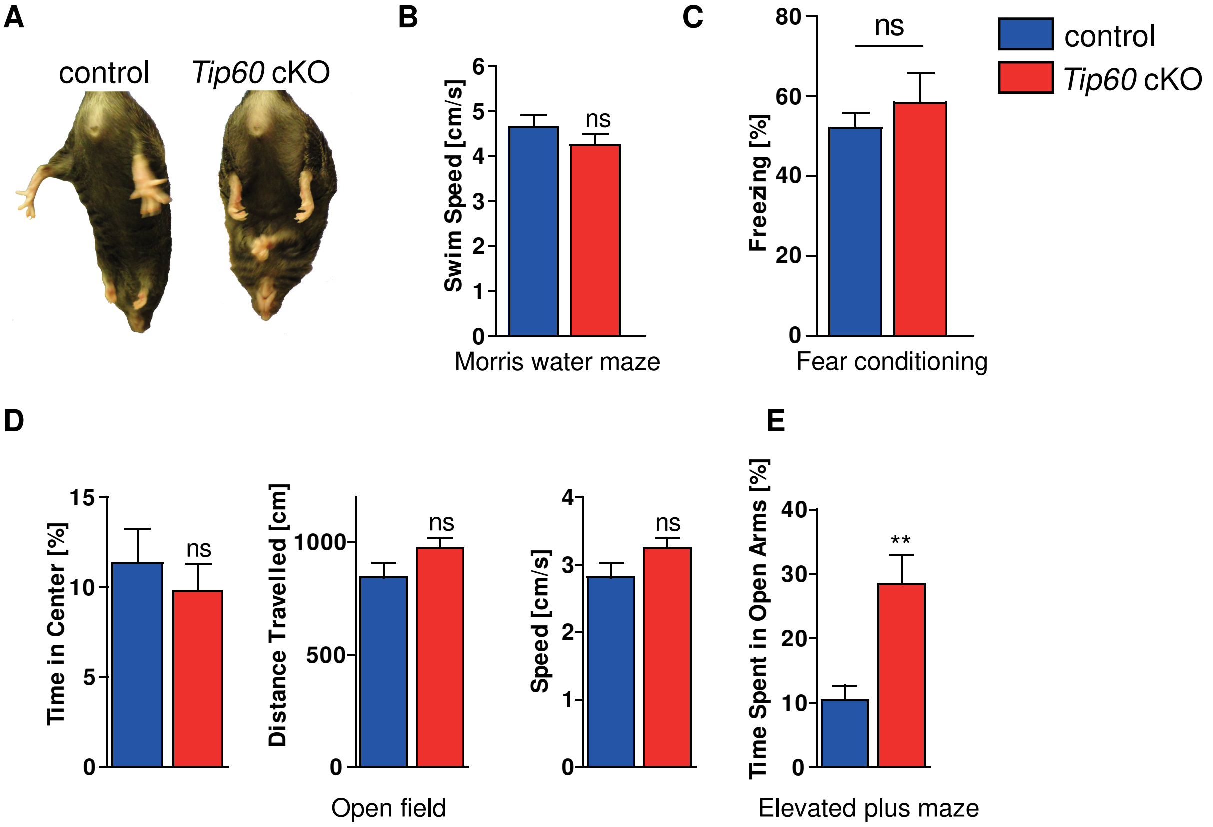

Figure S7: Behavior.

(A) A *Tip60* cKO animal shows limb clasping at 4 months after tamoxifen injections. No limb clasping is observed in the control animal.

(B) Morris water maze. Average swim speed was comparable between *Tip60* cKO and control animals.

(C) Contextual fear conditioning. *Tip60* cKO and control mice showed comparable freezing behavior during the memory test (ns, $p > 0.05$), n = 10-12.

(D) The percentage of time spent in the center of the open field arena was similar between control and *Tip60* cKO mice during a 5 min trial (left). There were no differences in the distance travelled (middle) or the speed (right) between genotypes.

(E) The percentage of time spent in the open arms of the elevated plus maze was significantly higher in *Tip60* cKOs.

Two-tailed student's t-test, ns: $p > 0.05$, *$p < 0.05$, **$p < 0.01$. Error bars represent SEM. n = 9-13 per genotype.

SUPPLEMENTARY METHODS

***In situ* Hybridization (ISH)**

Probes used were:

Lin7b	NM_011698514, nts 12–525, length 514 bp
Esr1	NM 007956, nts 1034–1998, length 964 bp
Cacng8	NM_133190, nts 222–713, 492 bp
Syt7	NM_018801, nts 446–1479, length 1034 bp
Ryr1	NM_009109.1, nts 13752–14519 length 798 bp
Syt4	NM_009308, nts 328–1305, length 977 bp
Marcksl1	NM_010807.4, nts 346–1289, length 944 bp
Syt5	NM_016908, nts 35–934, length 899 bp

Quantitative Real-time PCR

Primers used were (5'->3'):
Gap43 forward: AGA TGG TGT CAA GCC GGA AG
Gap43 reverse: CGC CTT TGA GCT TTT TCC TTG T
Syt5 forward: TGG GCC GGA GTT ACA TAG ATA A
Syt5 reverse: GGC CTA GCT GGT GTT TGT CTG
Syt17 forward: GAC ATC AAA CCC GTT GAG TTC G
Syt17 reverse: GTC ATC GGG CGT GTA GGT C
Sst forward: ACC GGG AAA CAG GAA CTG G
Sst reverse: TTG CTG GGT TCG AGT TGG C
Syt9 forward: CTG CCA AGA TTT CAT CTA CCA CC
Syt9 reverse: TCC AAG ACA CGA AAA GAG AGA CA
Sphkap forward: GAG CGG TCC ATG AGT GAA TTA G
Sphkap reverse: CAG AGG CAT GTT CCT TTA TGC T
Sirpa forward: GCT ACC CAC AAC TGG AAT GTC
Sirpa reverse: GGT TAT TTC CCT GGC GTT CTT
Orai1 forward: GAT CGG CCA GAG TTA CTC CG
Orai1 reverse: TGG GTA GTC ATG GTC TGT GTC
Gapdh forward: CAT GGC CTT CCG TGT TCC TA
Gapdh reverse: CCT GCT TCA CCA CCT TCT TGA
Stmn3 forward: CAG CAC CGT ATC TGC CTA CAA
Stmn3 reverse: GTA GAT GGT GTT CGG GTG AGG
Dnm1 forward: AAT ATG CCG AGT TCC TGC ACT
Dmn1 reverse: GTC TCA GCC TCG ATC TCC AG
Stau2 forward: CGA AGG GAG CAG TAT AAA GAA GG
Stau2 reverse: GGT TTG GGA AGC GTA GAT TCA G
Thy1 forward: TGC TCT CAG TCT TGC AGG TG
Thy1 reverse: TGG ATG GAG TTA TCC TTG GTG TT
Ywhah forward: ACG AAG ATC GAA ATC TCC TCT CT
Ywhah reverse: CCG GTA GGC TTT AAC TTT CTC CA
Cdc20 forward: GCC GAA CTC CTG GCA AAT CTA
Cdc20 reverse: TTG GGG GAT AAA GCG GTC AC
Pole forward: CTT TGG AGC GTC GCA ATG G
Pole reverse: ATC TCA GTA GGG TGC ATG TTG AT
Zranb3 forward: GCC ACC TTT CCC ACG AGT AG
Zranb3 reverse: TAG GCA TGG TGA GCC ACA TC
Dtdt1 forward: GGC TAG CGT CAC AGT TGG AG
Dtd1 reverse: CTG ACA CAC AGC ACC TCG TA
Mad2l1 forward: CGA GAC GGT TCG TTT GGT CT
Mad2l1 reverse: ACC GTC TCT TCA TTG ATG GGG
Adcyap1 forward: CTG CGT GCA GAA ATG CTA CTG
Adcyap1 reverse: AGC CGT AGA GTA ATG GTG GAT AG
Ryr1 forward, reverse: Mm_Ryr1_1_SG QuantiTect Primer (Qiagen)
Egr2 forward, reverse: Mm_Egr2_1_SG QuantiTect Primer (Qiagen)
Esr1 forward, reverse: Mm_Esr1_2_SG QuantiTect Primer (Qiagen)
Crh forward, reverse: Mm_Crh1_1_SG QuantiTect Primer (Qiagen)
Lin7b forward, reverse: Mm_Lin7b_1_SG QuantiTect Primer (Qiagen)

Reln forward, reverse: Mm_Reln_1_SG QuantiTect Primer (Qiagen)
